# Development and Preliminary Validation of a Novel Protein Assessment Screening Tool (PAST) for Nutrition Care

**DOI:** 10.3390/nu18142378

**Published:** 2026-07-21

**Authors:** Allison T. Contillo, Ock K. Chun, Nancy R. Rodriguez

**Affiliations:** Department of Nutritional Sciences, University of Connecticut, Storrs, CT 06269, USAock.chun@uconn.edu (O.K.C.)

**Keywords:** diet screener, protein, healthy aging, perioperative nutrition, joint arthroplasty

## Abstract

**Background/Objectives.** Protein needs increase with aging to support muscle health. These needs are further increased in older adults undergoing surgery, particularly in procedures limiting acute post-operative activity, such as total joint arthroplasty (TJA). However, tools to efficiently characterize dietary protein intake in clinical settings have remained undeveloped. This report describes the initial development and preliminary validation of a novel protein screener (Protein Assessment Screening Tool, PAST) for community-dwelling adults and a clinical population undergoing elective total joint arthroplasty (TJA). **Methods.** Participants (n = 48; 33 female, 15 male) were aged 40–70 years in the community-dwelling group (n = 22) and aged 50–70 years in the elective TJA group (n = 26). Both groups completed a 3-day food record and the PAST at two timepoints. Descriptive statistics were performed on demographic data. Differences between male and female participants and between groups were determined using independent t-tests or Mann–Whitney U tests as appropriate. The screener and 3-day food records were compared to determine sensitivity, specificity, and negative and positive predictive values (NPVs and PPVs), with a threshold of ≥1.2 g/kg/day representing adequate protein intake. **Results.** The PAST had relatively high sensitivity (78–87%) and PPVs (68–79%) but lower and more variable specificity (25–57%) and NPVs (33–57%); however, the 95% confidence intervals indicated variability. **Conclusions.** These preliminary findings demonstrate the potential clinical utility of the PAST. However, additional research is required to validate this tool prior to broad implementation in clinical practice.

## 1. Introduction

Guidelines for healthy aging [1] and perioperative nutrition [2,3] encourage elevated protein intakes (≥1.2 g/kg/day) for maintenance of muscle mass, strength, and function (i.e., muscle health). Therefore, an important clinical directive includes targeting and promoting higher protein intake to improve muscle health outcomes, particularly in older adults undergoing elective surgeries for whom adequate protein intake could optimize pre-operative nutrition status. Characterizing protein intake with traditional diet assessment tools, such as a 24 h recall or food record, can be challenging in clinical settings given the time required for subsequent nutrient analyses [4]. Briefer tools, such as a protein-focused diet screener, overcome the limitation of time in exchange for less accuracy and precision [4], emphasizing the importance of thoughtful design and development of an instrument to effectively estimate protein intake and characterize dietary adequacy in clinical populations.

Currently available protein-focused diet screeners have not been validated in clinical populations, are limited in the number of protein-specific food options, and do not consider routine consumption of protein-containing supplements [5,6]. The latter may be routinely consumed by community-dwelling older adults or recommended during the perioperative period to effectively increase total protein intake in these populations. A wider variety of food sources and their respective serving sizes should be considered in efforts to estimate protein intake and possibly characterize protein quality, both of which play a major role in the maximal stimulation of muscle protein synthesis (MPS) [7,8]. Therefore, the development and preliminary validation of a contemporary rapid protein screening tool in older individuals were undertaken in the present research initiative.

Screener development requires multiple stages prior to implementation in a clinical setting [9]. While the finite food list in a screener saves time by limiting assessment to relevant protein-rich foods, not assessing the diet in its entirety could lead to skewed results on protein intake. Additionally, with all screening tools, inappropriate or confusing wording for a question could lead to misrepresentation of nutrient intakes [10]. During the development process, receiving feedback from individuals representative of the intended population reduces some of the error inherent in diet screeners. This is accomplished through cognitive interviews that allow researchers to determine whether (1) the questions on the screener will secure appropriate information, (2) this tool is usable, and (3) whether the items assessed are appropriate. Following cognitive interviews, validating the instrument against an accurate reference tool at multiple timepoints will characterize the screener’s accuracy and precision. The validation process is essential to determine the screener modifications needed to improve, in this instance, the estimation of protein intake [9].

The objective of this work was to conduct a preliminary validation of a newly developed and novel protein-focused screening instrument, the Protein Assessment Screening Tool (PAST), in older adults who were either community-dwelling or receiving elective total joint arthroplasty (TJA).

## 2. Materials and Methods

*Screener development.* We previously published a model for screener development [9] that outlines the process used to develop the PAST. The PAST intends to assess the consumption frequency and portion sizes of foods and beverages, including common protein supplements, that routinely contribute significant amounts of protein to the American diet over a one-week timeframe. A copy of the PAST can be found as a Supplementary Material. The calorie and protein contents for each food item were determined using the USDA National Nutrient Database for Standard Reference available on the Nutritionist Pro^TM^ software (Nutritionist Pro^TM^, Axxya; Redmond, WA 98073, USA). For questions assessing multiple foods, the most commonly consumed food items were chosen for question coding (e.g., “red meat” was coded as 85% lean ground beef).

Following IRB approval, a cognitive interview pilot study (n = 9) was performed in healthy adults aged 40–70 years who were able to read and write in English. Written informed consent was obtained at the start of each individual interview. Two interview rounds were conducted to test the feasibility of the screener and obtain feedback from a similar research demographic planned for use in the validation studies. Each round was followed by modifications based on feedback. Given this study’s exploratory nature, this pilot study concluded following the second interview round, as researchers had obtained consistent feedback among participants and no additional alterations were identified for this tool. This finalized version of the screener was created on the Qualtrics and Research Electronic Data Capture (REDCap) platforms for use in validation studies in a community-dwelling population and a clinical population undergoing TJA. The current version of the screener is copyrighted (2023) through the University of Connecticut (UConn).

*Validation studies.* Community-dwelling group: Following UConn IRB approval, community-dwelling participants were recruited online through the UConn Storrs Faculty and Staff Daily Digest and via flyers posted on the UConn Storrs campus and in the nearby Mansfield community. Participants were required to be aged 40–70 years and needed to be able to read and write in English. The community-dwelling participants received a link to an information sheet before confirming their intent to enroll in this study; as this study posed minimal risk to participants, informed consent was waived. After enrollment, the community-dwelling participants were assessed at two timepoints (i.e., baseline and two weeks later), with a similar time frame used for the clinical group. These individuals comprised the control group for assessment alongside the clinical group [11].

Clinical group: Following dual approval from the UConn and Hartford Healthcare (HHC) IRBs, individuals aged 50–70 years for the TJA group (n = 26) were identified through the Epic clinic schedule (Epic Systems Corporation, Verona, WI, USA) at the HHC Bone and Joint Institute’s (HHC-BJI) Procedure-Related Education and Pre-Anesthesia Risk Evaluation (PREPARE) Center. The TJA participants were either undergoing elective total knee arthroplasty (TKA, n = 10) or total hip arthroplasty (THA, n = 16) due to osteoarthritis. Individuals were excluded if they had BMIs > 35 kg/m^2^, were unable to read and write in English, had prior TKAs or THAs, or were receiving surgery for a reason other than osteoarthritis.

Researchers met in person with eligible participants at the PREPARE Center to confirm eligibility and discuss study requirements. Eligible and interested participants provided written informed consent during the meeting. After enrollment, the TJA group was assessed at two timepoints: pre-operatively (between recruitment and day of surgery) and 1–4 weeks post-operation. At the first timepoint, the participants completed a short demographics questionnaire and then completed the PAST. On the same day, the participants started the 3-day food record (which was submitted electronically or as a hard copy). The participants completed an additional copy of the PAST and another 3-day food record post-operatively. Timeframes for the dietary assessments in the clinical group differed slightly from the community-dwelling participants (2–4 weeks post-operatively vs. 2 weeks, respectively) because the post-operative timepoint was determined by the HHC PREPARE staff in consultation with clinical participants.

*Dietary analyses.* All food records were analyzed using Nutritionist Pro^TM^ (Nutritionist Pro^TM^, Axxya; Redmond, WA 98073, USA). The PAST’s ability to identify those at risk of inadequate protein intake (defined as <1.2 g of protein/kg/d [1]) was evaluated against respective 3-day food records. Three-day food records, a conventional dietary assessment instrument, were chosen as a reference to validate the PAST while minimizing participant burden during the perioperative period. Height and weight were self-reported on the food record. A researcher who was also a registered dietitian nutritionist (RDN) contacted participants directly if clarification was needed for information provided on the food records.

*Statistical Analyses.* Calculating an accurate power analysis for study recruitment was challenging given the limited number of published studies for protein-specific screeners and the preliminary nature of this work. Therefore, a rolling effect size analysis based on the O’Brien–Fleming method was employed, where results were analyzed for every 20 to 30 patients for the TJA group [12]. The sample size for the community-dwelling group was based on a similar study by Morin et al. [5].

Statistical analyses were performed using SPSS software (IBM SPSS Statistics, Version 28, Chicago, IL 60606, USA) and an alpha level set at *p* ≤ 0.05. Descriptive statistics were used to summarize demographic information. Shapiro–Wilk tests were used to determine normality for demographic data; all demographic data (except for age for males in the TJA group) were normally distributed and were analyzed using independent t-tests to compare male and female participants within and between groups. Ages within the TJA group and for males between groups were analyzed using Mann–Whitney U tests.

To characterize the ability of the protein screener to accurately categorize participants as consuming adequate or inadequate protein intake, the sensitivity, specificity, and positive and negative predictive values (PPVs and NPVs) of the instrument were evaluated using the food record as the reference tool. The following categorizations [13] were used:

1. True negative (TN): adequate protein intake on the screener and on the food record (i.e., “true adequate consumer”)
2. True positive (TP): inadequate protein intake on the screener and on the food record (i.e., “true under consumer”)
3. False negative (FN): adequate protein intake on the screener, inadequate protein intake on the food record (i.e., “false adequate consumer”)
4. False positive (FP): inadequate protein intake on the screener, adequate protein intake on the food record. (i.e., “false under consumer”)

Sensitivity reflects the ability of the screener to correctly determine participants consuming inadequate protein among only those who consumed inadequate protein (as determined by the food record) and was calculated as [13]

[TP/(TP + FN)] × 100

Specificity reflects the ability of the screener to correctly determine participants consuming adequate protein among only those who consumed adequate protein (as determined by the food record). This was calculated as [13]

[TN/(FP + TN)] × 100

The PPV reflects the ability of the screener to identify participants consuming inadequate protein intake from all individuals that might or might not have adequate protein intake. This was calculated as [13]

[TP/(TP + FP)] × 100

The NPV reflects the ability of the screener to identify participants consuming adequate protein intake among all individuals that might or might not have adequate protein intake. This was calculated as [13]

[TN/(FN + TN)] × 100

Calculations were reported as whole numbers (for the numbers of TNs, FNs, TPs, and FPs) or percentages (the calculations above) [13]. The 95% confidence intervals (95% CIs) were calculated using MedCalc online program (available at https://www.medcalc.org/en/calc/diagnostic_test.php, accessed 19 June 2026).

## 3. Results

### 3.1. Demographics

Table 1 shows the demographic information for the 48 participants (22 community-dwelling, 26 TJA; 33 female, 15 male) who completed at least one copy of the screener and food record at the same time point. For the community-dwelling adults, ages, weights, and BMIs were similar between the sexes; however, male participants were taller than female participants (mean difference (MD): −13.3 (cm); standard error (SE): 3.9; 95% confidence interval (95% CI): [−21.5, −5]; *p* < 0.01). In the TJA group, age and BMI were similar between the sexes; however, the male participants were taller than in the community-dwelling group (MD: −15.8 (cm); SE: 3.1; 95% CI: [−22.1, −9.4]; *p* < 0.001) and weighed more (MD: −19.2 (cm); SE: 5.3; 95% CI: [−30.2, −8.2]; *p* < 0.001).

There were no between-group differences in weight, height, or BMI for the female participants. The female participants in the TJA group were older than those in the community-dwelling group (MD: 11.3; SE: 2.4; 95% CI [6.4, 16.2]; *p* < 0.001). The male participants in both groups were similar in height. The males in the TJA group weighed more (MD: 19.2; SE: 6.9; 95% CI [4.3, 34.2]; *p* < 0.05); as a result, the males in the TJA group also had a higher average BMI (MD: 5.5; SE: 1.1; 95% CI [3, 8]; *p* < 0.001).

### 3.2. Sensitivity, Specificity, PPVs and NPVs

Table 2 shows the screener categorization of each individual at each time point and the resulting sensitivity, specificity, PPVs and NPVs. Among all participants, the screener scored relatively high on sensitivity (78–87%) and PPVs (68–79%), and overall low to moderate on specificity (25–57%) and NPVs (33–57%) (Table 2).

## 4. Discussion

In practice, nutrition screening utilizes various tools to determine malnutrition risk [14]. Protein intake, a critical macronutrient for recovery and a known contributor to muscle health, is not specifically targeted in these instruments. Further, because elective surgery would be delayed in a malnourished patient, increasing protein intake to the recommended level of ≥1.2 g/kg/day [1,2] would be a relevant nutrition intervention for older adults and incoming patients undergoing elective TJA. The ability to characterize protein intake as adequate or inadequate distinguishes the PAST from other protein-specific dietary screening tools and provides a foundation for its clinical and practical utility [6,15,16]. The preliminary validation conducted in the present paper demonstrates that a unique screening tool such as the PAST appropriately characterizes adequate vs. inadequate protein consumers, which would be useful for effective nutrition resource allocation in a clinical as well as community setting.

In this sample of community-dwelling adults and individuals receiving elective TJA, the PAST was found to have relatively high sensitivity (78–87%) and PPVs (68–79%) but lower and more variable specificity (25–57%) and NPVs (33–57%; ≤50% at two timepoints), although the 95% confidence intervals (particularly for specificity and NPVs) suggest variability (potentially due to limited sample size). For practical application of the screener, PPVs and NPVs are more valuable, as these values represent the screener’s ability to characterize protein intake in samples containing both under- and adequate consumers [13]. In this preliminary validation process, the PPV results suggest that in this sample, a positive result on the PAST appropriately characterized a majority of individuals as true protein under-consumers who would likely benefit from education and resources for increasing routine protein intake. Although low NPVs are a concern, the authors suspect that this reflects the sample characteristics rather than an issue with this tool. The NPV calculation considers the number of people who were appropriately or inappropriately categorized as consuming adequate protein (i.e., true or false adequate consumers); the number of participants in either category was low in this sample. This, along with the small sample size, contributed to the low-to-moderate NPVs [13]. The NPV-related findings are noteworthy because the low number of true adequate consumers serves as a call to action for nutrition education in this group. Additionally, the low total number of false adequate consumers (i.e., individuals that would be “missed” by the PAST and not receive additional intervention) demonstrates the utility of this tool. This initial development and validation of the PAST is encouraging and indicates that more extensive work in refining and validating the PAST would be worthwhile.

*Applications*. Optimally supporting muscle health (i.e., the maintenance and recovery of muscle mass, strength, and function) requires adequate protein intake [1,2,17]. Meeting protein recommendations for healthy aging contributes to maintenance of physical function during aging. McGrath et al. found that adults aged 60+ who met the lower end of the protein recommendation for healthy aging (≥1.0 g/kg/d) had lower risk of functional disability on a questionnaire about self-reported activities of daily living [18]. Although causation cannot be determined, the documented association between protein intake and physical function emphasizes the importance of addressing suboptimal protein intake in aging populations [18]. Although this work includes findings in community-dwelling and clinical populations, the intent is not to directly compare the groups (or this tool’s utility in one versus the other) but rather to highlight the utility of this tool in different aging populations. Special considerations for use in clinical care include post-operative pain and medication use (particularly immediately following surgery), which can impact someone’s ability to complete this tool. This is illustrated in the present study with the different follow-up timeframe for the groups (please see the Section 2 for additional information). The development of a protein-focused screening tool for older adults, such as the PAST, is particularly important in clinical settings given work showing suboptimal protein intakes in this population despite recommendations for increased protein to support post-operative recovery [17,19,20].

*Limitations and Future Research Areas.* The aim of this preliminary study was to develop and validate a novel protein-focused screening tool to rapidly characterize routine protein intake in an older clinical population. The limitations of the present study include the small sample sizes (n = 22, n = 26, control and TJA, respectively) and non-diverse participant demographics. The current findings on specificity, sensitivity, PPVs, and NPVs are preliminary and limited by the small sample size. Future work to validate this tool should determine the sample size based on the desired sensitivity and estimated prevalence of suboptimal protein intake for the surveyed population. Based on our findings, an estimated 80–100 or 270–300 individuals would be needed for more stable estimates of sensitivity and specificity, respectively. Increasing the sample size would likely narrow the 95% confidence intervals and improve the utility of this tool. Although demographics were not collected for the cognitive interview study, the community-dwelling participants were predominantly female (82%) and white (91%). Approximately 58% of the TJA group participants were female and all identified as white, non-Hispanic/Latino. Research has shown gender differences in estimating portion size and desired portion size [21], which may have impacted male participants’ completion of the PAST. Additionally, populations not represented by the sample may make different protein-containing food choices that were not assessed by the current version of the PAST, limiting applications to other populations where the screener may not be culturally appropriate. Although additional work is warranted in more diverse samples, the findings from this work provide a basis for refining the PAST for use in larger and more diverse populations of older adults through additional research.

Additionally, errors in self-reporting anthropometric data or dietary intake may have impacted screener classification. For example, errors in self-reported weight could have impacted the g/kg estimates of protein intake. Further, reporting errors are common on self-reported food records, negatively impacting the validation of the PAST and the resulting sensitivity, specificity, NPVs and/or PPVs. Follow-up studies to validate this tool could decrease self-reporting errors by collecting anthropometric measures in person, requiring participants to weigh consumed food, or using another method of verification (e.g., photos, random 24 h recalls, etc.). Participant burden should be considered with these protocol changes, particularly for adults recovering from surgery.

Future improvements to screener design and additional research implementing the PAST are also recommended to further improve PPVs and NPVs. For example, to minimize completion time, questions on protein intake from grains (pasta, breads, rice, etc.) were excluded based on their smaller protein contributions to the American diet [22]. While adding a proxy question for this food group (and/or other lesser consumed protein-containing foods) might improve estimates of protein intakes [23], such additions should be thoughtfully considered to maintain the brevity of this tool.

Our findings indicate that increasing sample size and administering this tool in samples with higher numbers of adequate protein consumers could advance improvements in NPVs (and also improve specificity by decreasing false positives). However, given the recommended intervention based on screener results (i.e., nutrition education), false under-consumers (i.e., “false positives”) are less of a concern than minimizing false adequate consumers (i.e., “false negatives”), who would require intervention but would be missed by the screener. Based on an individual’s result, identified patients could be referred to an RDN for nutrition education prior to elective orthopedic surgery, supporting perioperative care. Given that the vast majority of U.S. older adults have at least one chronic health condition [24], any false positives would likely still benefit from receiving nutrition education focused on meeting current recommendations for protein intake in older adults [25].

Screener interpretation may also impact the aforementioned values; for example, overestimating consumption frequency could lead to a falsely elevated protein intake result. Portion size estimations may also play a role, as accurate estimation is a common challenge when using diet screening and assessment tools [26]. The selections on the current screener were chosen to provide options below, at, and above standard consumption frequency and portion sizes to accommodate variations in the diet. If the highest portion size option was not high enough for someone to select their true portion size, this could lead to their categorization as a “false under-consumer”. However, participants selecting the maximum portion size was not a consistent finding for under-consumers in this sample, suggesting that this was not the sole or primary factor for any improper categorization. In the instances where under-consumers selected the highest portion size, meat and poultry were the most common categories; future iterations of this tool could include higher portion size options for this category. For modification to accommodate cultural differences in dietary habits, additional cognitive interviews should be implemented to ensure appropriate portion size and consumption frequency options [9,10,26]. In addition to increasing sample size, future work investigating whether adjustments to the timeframe used by the PAST (e.g., moving from 1 week to a 3-day period) or implementing other visuals/methods to estimate portion size (e.g., adjustments to photos or using videos) is warranted to advance this preliminary work.

Finally, the PAST provides an estimate of protein quantity relative to a 1.2 g/kg/day threshold. Because protein requirements are based on the true need for protein’s constituent essential amino acids (EAAs), insufficient EAA ingestion would be more likely if suboptimal protein intakes were sourced from lower-quality protein sources [27]. Therefore, both protein quantity and quality should be considered in assessment of adequate protein intake. Newer metrics, such as the EAA-9 and Meal Protein Quality Score (MPQS), provide valuable information on protein quality and, unlike traditional metrics, can characterize protein quality in the diet [28,29]. While these tools are promising, the need to obtain food record data presents a challenge to assessing protein quality in clinical settings, where the time for nutrition screening is limited and the brevity of a diet screener such as the PAST is likely more practical. Future work coupling the PAST with a protein quality measure would provide a more holistic measure of protein consumption and be a worthwhile endeavor.

## 5. Conclusions

The PAST demonstrated overall high sensitivity and PPVs (outside of the lower PPV in the TJA group at post-op). The latter suggests that individuals identified by the PAST are likely to consume inadequate protein. The current work demonstrates the potential clinical utility of a brief protein-focused tool for perioperative nutrition screening to identify inadequate protein consumers. These individuals could be directed to additional nutrition resources to achieve recommended protein intake and help optimize perioperative care and muscle health. However, further development of this tool, coupled with additional validation employing larger and more diverse populations, is warranted for the PAST to be effectively utilized in practice.

## Figures and Tables

**Table 1:** Demographic information for community-dwelling and total joint arthroplasty participants ^1,2^.

	Community-Dwelling (n = 22)		Total Joint Arthroplasty (n = 26)	
	Female (n = 18)	Male (n = 4)	Female (n = 15)	Male (n = 11)
Average Age (years)	52.7 ± 7.3	55 ± 11.7	64 ± 6.4 ^a^	64.5 ± 4.2
Height (meters)	1.6 ± 0.1	1.8 ± 0.1 ^b^	1.6 ± 0.1	1.8 ± 0.1 ^c^
Weight (kg)	70.6 ± 11.3	74.3 ± 6.8	74.3 ± 13.7	93.5 ± 12.9 ^c,d^
BMI (kg/m^2^)	26.2 ± 4.0	23.6 ± 1.0	27.9 ± 4.9	29.1 ± 3.5 ^a^
Race/Ethnicity				
Non-Hispanic White (%)	-	-	100%	100%

^1^ Total joint arthroplasty (TJA). ^2^ Values are means ± standard deviation (SD). ^a^ Higher in the TJA group within sex; *p* < 0.001. ^b^ Higher in males vs. females within group; *p* < 0.05. ^c^ Higher in males vs. females within group; *p* ≤ 0.001. ^d^ Higher in the TJA group within sex; *p* < 0.05.

**Table 2:** Sensitivity, specificity, and positive and negative predictive values of the protein screener at various time points for community-dwelling and total joint arthroplasty participants ^1,2^.

	Community-Dwelling		Total Joint Arthroplasty	
	Timepoint 1 (n = 21)	Timepoint 2 (n = 13)	Pre-Op (n = 24)	Post-Op (n = 23)
True under-consumers	11	7	14	13
True adequate consumers	4	1	2	2
False under-consumers	3	3	5	6
False adequate consumers	3	2	3	2
Sensitivity (95% CI)	79% (49.2, 95.3)	78% (40.0, 97.2)	82% (56.6, 96.2)	87% (59.5, 98.3)
Specificity (95% CI)	57% (18.4, 90.1)	25% (0.63, 80.6)	29% (3.67, 71.0)	25% (3.19, 65.1)
PPV (95% CI)	79% (59.9, 90.0)	70% (54.6, 81.9)	74% (62.5, 82.5)	68% (58.1, 77.2)
NPV (95% CI)	57% (28.8, 81.4)	33% (5.81, 80.2)	40% (12.3, 76.0)	50% (14.7, 85.4)

^1^ PPV: positive predictive value; NPV: negative predictive value. ^2^ Timepoint 1: baseline; Timepoint 2: 2 weeks following the baseline measure; Pre-Op: between recruitment and day of surgery; Post-Op: 1–4 weeks post-operation.

## Data Availability

Permission for data sharing was not obtained during the informed consent process; as a result, this data is not available publicly. Further inquiries on the PAST or additional information can be directed to the corresponding authors.

## Supplementary Materials

The following supporting information can be downloaded at: https://www.mdpi.com/article/10.3390/nu18142378/s1. Supplementary Materials: Protein Screener.

## Abbreviations

The following abbreviations are used in this manuscript:

PAST	Protein Assessment Screening Tool
TJA	Total joint arthroplasty
NPV	Negative predictive value
PPV	Positive predictive value
MPS	Muscle protein synthesis
REDCap	Research Electronic Data Capture
UConn	University of Connecticut
HHC	Hartford Healthcare
HHC-BJI	Hartford Healthcare Bone and Joint Institute
PREPARE	Procedure-Related Education and Pre-Anesthesia Risk Evaluation
TKA	Total knee arthroplasty
THA	Total hip arthroplasty
RDN	Registered dietitian nutritionist
TN	True negative
TP	True positive
FN	False negative
FP	False positive
MD	Mean difference
SE	Standard error
EAA	Essential amino acid
MPQS	Meal Protein Quality Score
