# Supplementary material for: Development and Preliminary Validation of a Novel Protein Assessment Screening Tool (PAST) for Nutrition Care
Source: Nutrients. 2026 Jul 21;18(14):2378. doi: 10.3390/nu18142378 (PMC13416055; doi:10.3390/nu18142378)
Supplement: Supplementary file 1 [file nutrients-18-02378-s001.zip › nutrients-4367351-supplementary.pdf]

Protein Screener

AAA

1) **Please enter your participant ID number.**

2) **Please enter your weight in pounds.**
* must provide value

Please estimate the how often (frequency) and how much (portion size) you eat or drink each item ***during a typical week***.

For the frequency, select the circle that best reflects your answer.

For the amounts, please select the average amount that you would eat or drink in one sitting by selecting the circle that best reflects your answer.

When you select "Never" for any item, please select "N/A" for the portion size.

One serving (3 ounces) of meat, poultry, fish, or tofu is the size of the palm of your hand.

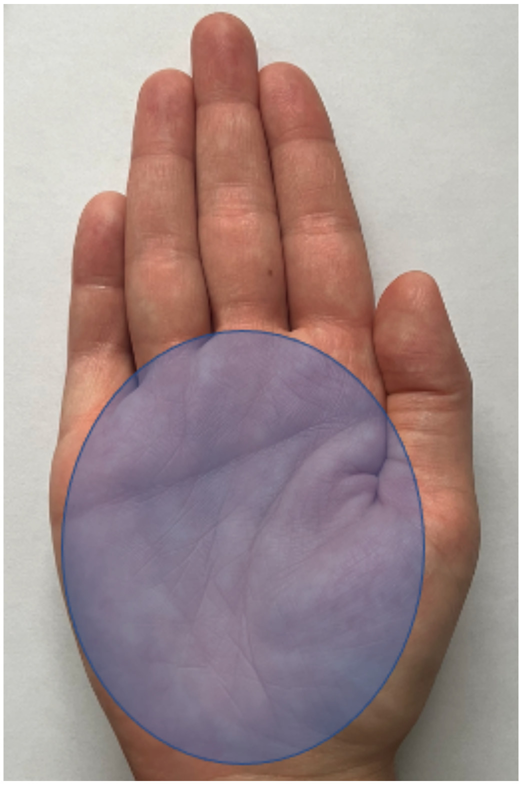

Please select the how often you eat meat, poultry, fish, or tofu below ***during a typical week.***
This includes the meat portions of dishes such as soups, sauces, or combination foods (such as

© 2023 University of Connecticut

lasagna or pizza).
Please use the photo above to help you estimate the number of servings you eat during one sitting

	Frequency							Servings				
	Never	1-2 times per week	3-4 times per week	5-6 times per week	Daily	2 times per day	≥ 3 times per day	N/A	0.5	1	1.5	2
Red meat (beef pork, lamb, etc.)	○	○	○	○	○	○	○	○	○	○	○	○
Poultry (chicken, turkey, etc.)	○	○	○	○	○	○	○	○	○	○	○	○
Fish/shellfish (salmon, shrimp, etc.)	○	○	○	○	○	○	○	○	○	○	○	○
Tofu	○	○	○	○	○	○	○	○	○	○	○	○

One serving (1/2 cup) of legumes, such as beans or chickpeas, is the size of a cupped handful.

© 2023 University of Connecticut

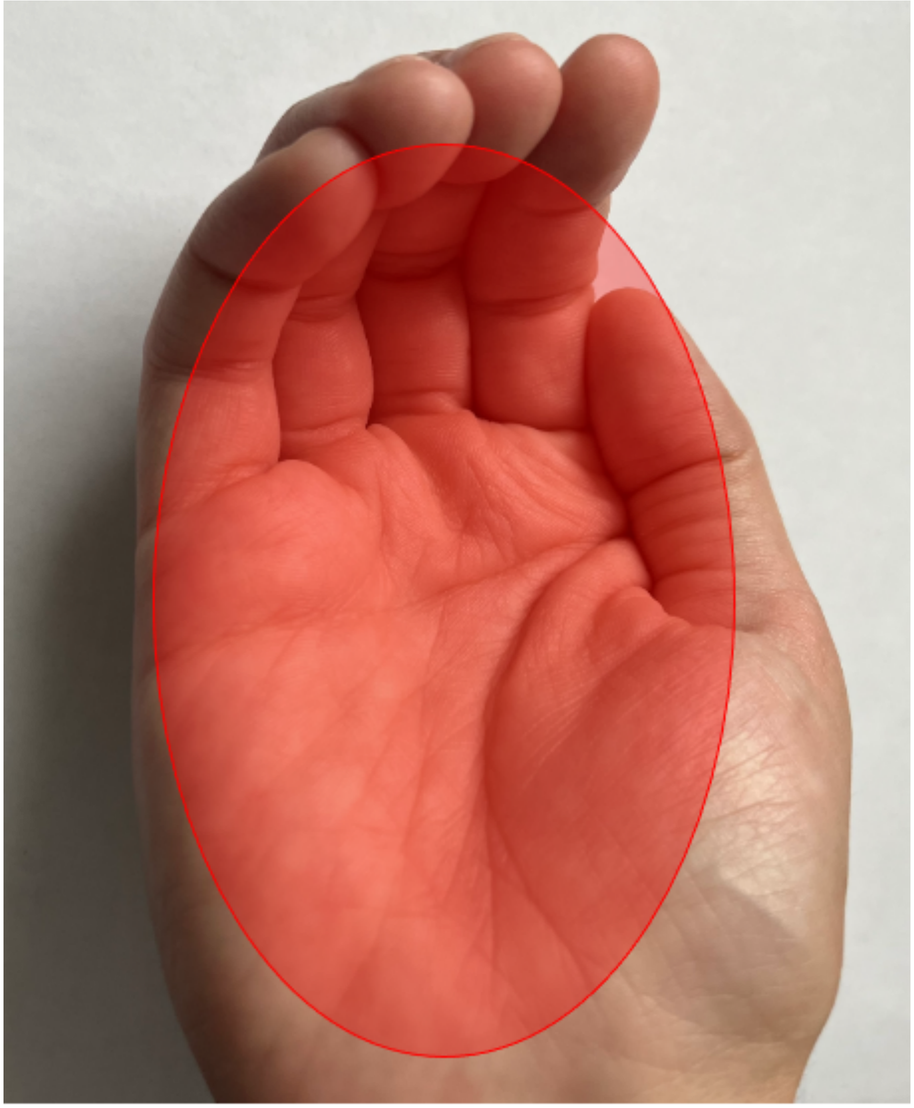

Please select the how often you eat legumes below ***during a typical week***.
Please use the photo above to help you estimate the number of servings you eat during one sitting.

	Frequency							Servings				
	Never	1-2 times per week	3-4 times per week	5-6 times per week	Daily	2 times per day	≥ 3 times per day	N/A	0.5	1	1.5	2
Legumes (black beans, chickpeas, etc.)	○	○	○	○	○	○	○	○	○	○	○	○

A serving (1 cup) of yogurt, Greek yogurt, or cottage cheese is one single-serving (~6 ounce) container, or the size of a fist.

© 2023 University of Connecticut

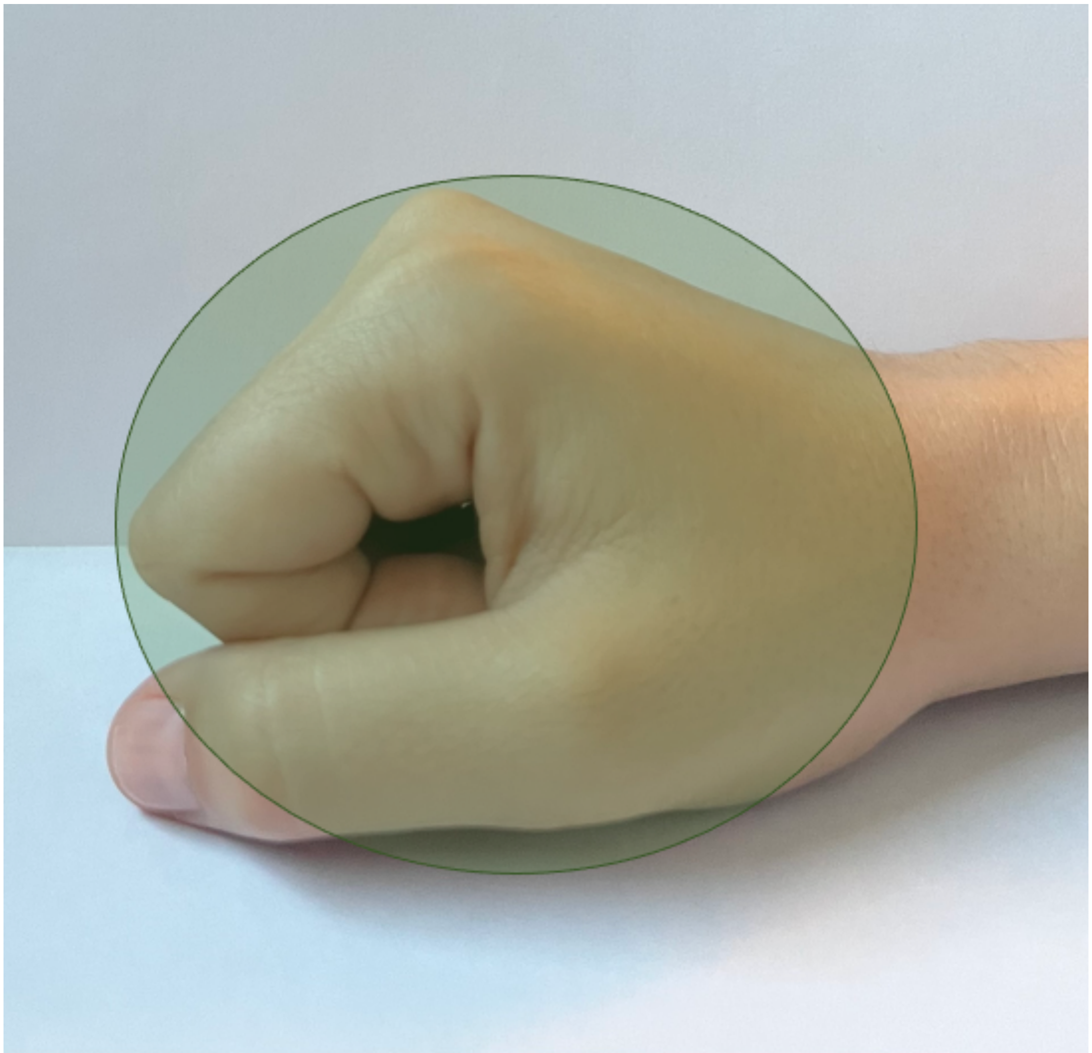

Please select the how often you eat yogurt, Greek yogurt, or cottage cheese below ***during a typical week***.
Please use the photo above to help you estimate the number of servings you eat during one sitting.

	Frequency							Servings				
	Never	1-2 times per week	3-4 times per week	5-6 times per week	Daily	2 times per day	≥ 3 times per day	N/A	0.5	1	1.5	2
Regular yogurt (NOT including Greek yogurt)												
Greek yogurt												

© 2023 University of Connecticut

Cottage cheese												

One serving of milk is around 1 cup (8 ounces), about the size of a baseball.

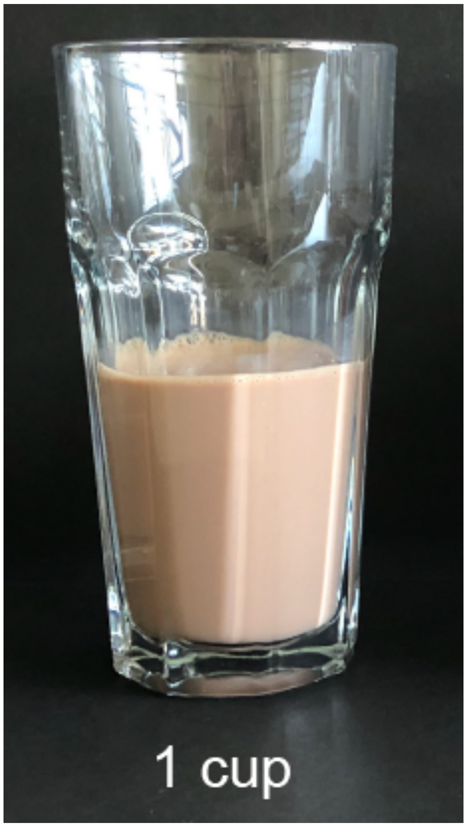

Please select how often you drink each beverage item ***during a typical week***.
Use the above photo of the cup to determine the average portion size that you drink in one sitting.

	Frequency							Servings				
	Never	1-2 times per week	3-4 times per week	5-6 times per week	Daily	2 times per day	≥ 3 times per day	N/A	0.5	1	1.5	2
Cow's milk												
Soy-based beverage												
Alternative milk beverage (NOT including soy,												

© 2023 University of Connecticut

including almond, oat, etc.)		

A serving of cheese is 1 ounce.
One ounce equals:
One slice,
or one cheese stick,
or the size of a golf ball (for shredded cheese),
or the size of 4 dice (for cheese from a block).

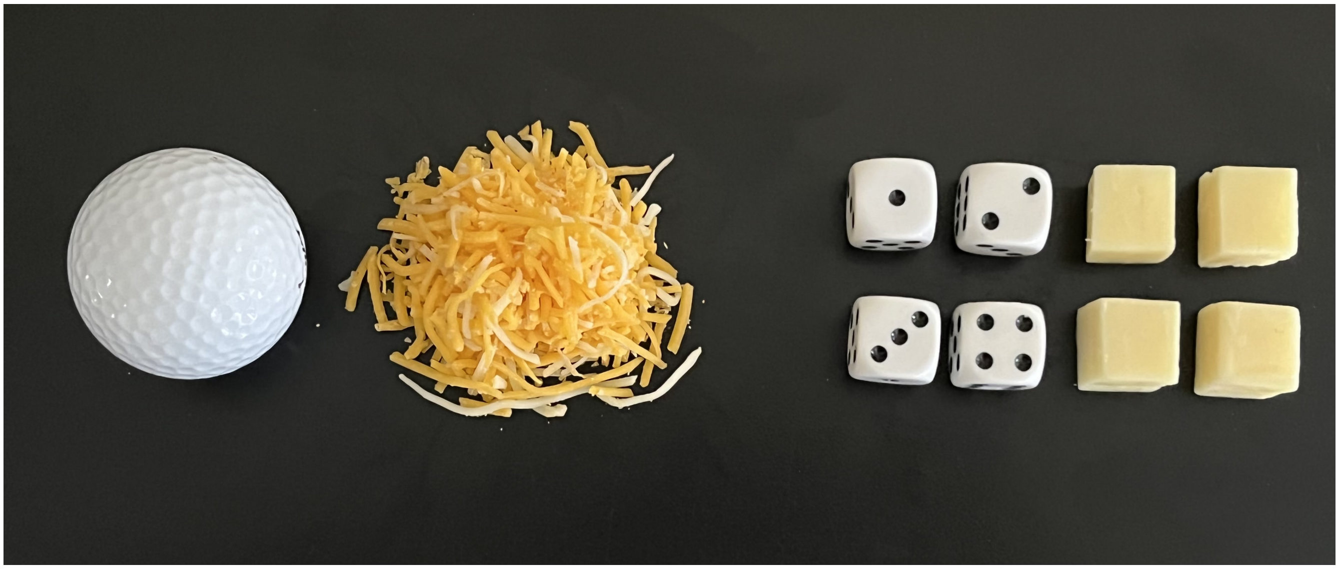

Please select the how often you eat cheese below ***during a typical week***.
Please use the photo above to help you estimate the number of servings you eat during one sitting.

	Frequency							Servings				
	Never	1-2 times per week	3-4 times per week	5-6 times per week	Daily	2 times per day	≥ 3 times per day	N/A	0.5	1	1.5	2
Cheese	○	○	○	○	○	○	○	○	○	○	○	○

A serving of peanut butter is 2 tablespoons.

© 2023 University of Connecticut

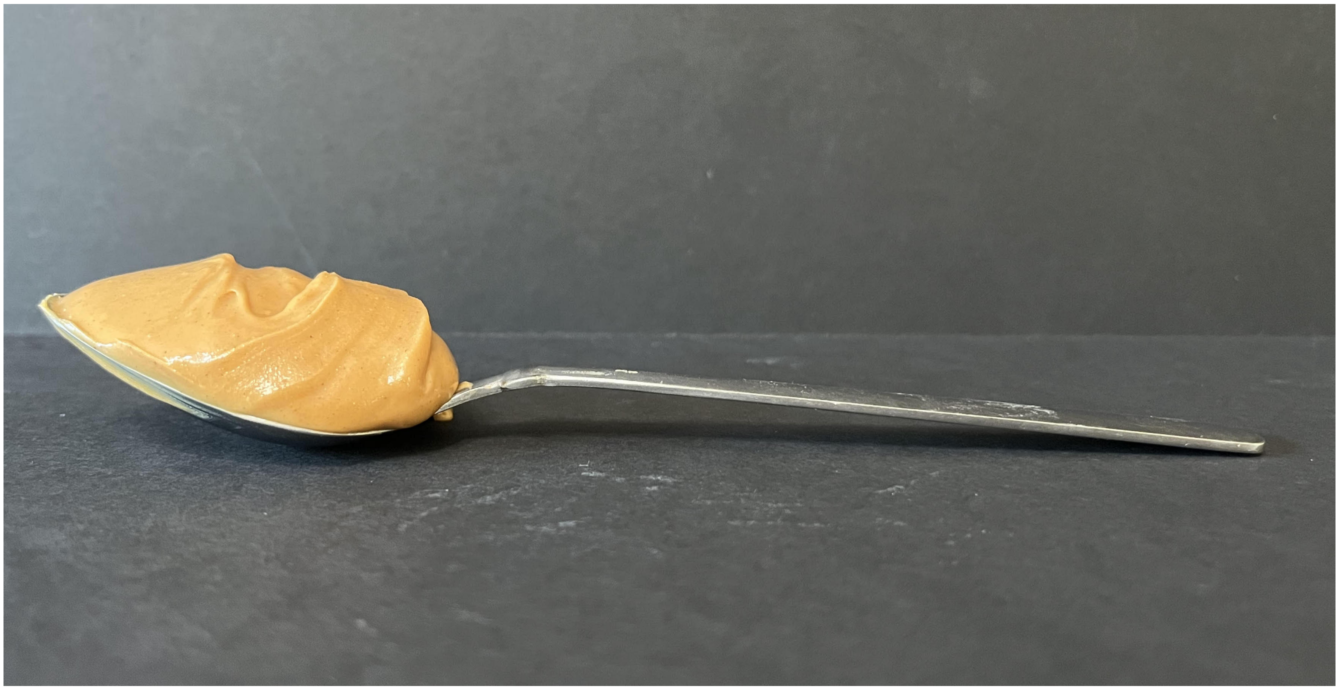

Please select how often you eat peanut butter or nut butter below ***during a typical week***.
Please use the photo above to help you estimate the number of servings you eat during one sitting.

	Frequency							Servings				
	Never	1-2 times per week	3-4 times per week	5-6 times per week	Daily	2 times per day	≥ 3 times per day	N/A	0.5	1	1.5	2
Peanut/nut butter	○	○	○	○	○	○	○	○	○	○	○	○

One serving (1/4 cup or 1 ounce) of nuts is around the size of a golf ball.
One ounce of nuts equals:
14 Walnut Halves,
or 24 Almonds,
or 35 Peanuts,
or 47 (shelled) Pistachios

© 2023 University of Connecticut

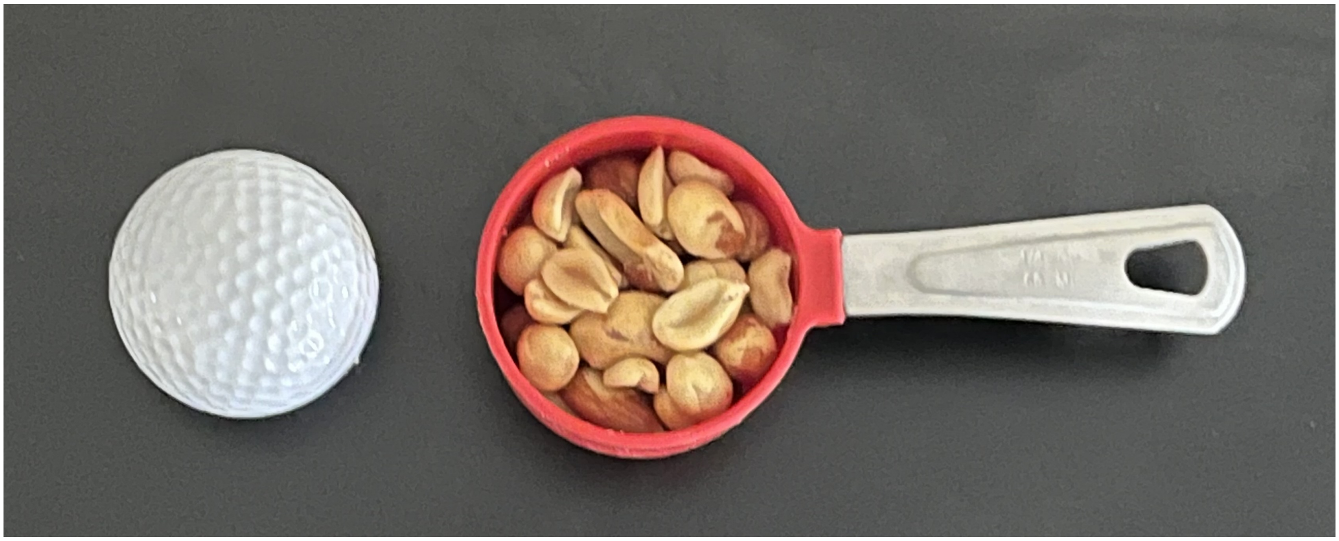

Please select how often you eat peanuts/nuts below ***during a typical week***.
Please use the photo above to help you estimate the number of servings you eat during one sitting.

	Frequency							Servings				
	Never	1-2 times per week	3-4 times per week	5-6 times per week	Daily	2 times per day	≥ 3 times per day	N/A	0.5	1	1.5	2
Peanuts/nuts NOT including peanut/nut butter	○	○	○	○	○	○	○	○	○	○	○	○

Please select how often you eat cold cuts (prepackaged or deli lunch meat) ***during a typical week.***
One serving (3 ounces) equals:
3 slices of prepackaged cold cuts
or 2 slices of deli-sliced cold cuts

	Frequency							Servings				
	Never	1-2 times per week	3-4 times per week	5-6 times per week	Daily	2 times per day	≥ 3 times per day	N/A	0.5	1	1.5	2
Cold cuts	○	○	○	○	○	○	○	○	○	○	○	○

Please select how often you eat eggs ***during a typical week***.
One serving equals one large egg.

© 2023 University of Connecticut

	Frequency							Servings				
	Never	1-2 times per week	3-4 times per week	5-6 times per week	Daily	2 times per day	≥ 3 times per day	N/A	1 egg	2 eggs	3 eggs	4 eggs
Eggs	○	○	○	○	○	○	○	○	○	○	○	○

Please select how often you drink ready to drink protein shakes ***during a typical week***.
Examples include pre-mixed protein shakes, such as Boost® or Ensure®.

	Frequency							Servings				
	Never	1-2 times per week	3-4 times per week	5-6 times per week	Daily	2 times per day	≥ 3 times per day	N/A	0.5 bottle	1 bottle	1.5 bottles	2 bottles
Ready to drink protein shakes	○	○	○	○	○	○	○	○	○	○	○	○

Please select how often you consume protein powders ***during a typical week***.
One serving equals 1-2 scoops depending on the protein powder you use.

	Frequency							Servings				
	Never	1-2 times per week	3-4 times per week	5-6 times per week	Daily	2 times per day	≥ 3 times per day	N/A	0.5	1	1.5	2
Protein powder	○	○	○	○	○	○	○	○	○	○	○	○

Please select how often you eat granola bars ***during a typical week***.
Examples of granola bars include Nature Valley®, Kind®, or a Clif Bar® containing ~5-10 grams of protein per bar.

	Frequency							Servings				
	Never	1-2 times per week	3-4 times per week	5-6 times per week	Daily	2 times per day	≥ 3 times per day	N/A	0.5 bar	1 bar	1.5 bars	2 bars
Granola bars	○	○	○	○	○	○	○	○	○	○	○	○

© 2023 University of Connecticut

Please select how often you eat protein bars ***during a typical week***.
Examples of protein bars include Powerbar®, Pure Protein®, or Quest® bars containing ~20 grams of protein per bar.

	Frequency							Servings				
	Never	1-2 times per week	3-4 times per week	5-6 times per week	Daily	2 times per day	≥ 3 times per day	N/A	0.5 bar	1 bar	1.5 bars	2 bars
Protein bars	◯	◯	◯	◯	◯	◯	◯	◯	◯	◯	◯	◯

Submit

Powered by REDCap

© 2023 University of Connecticut
